# NFAT2 Regulates Generation of Innate-Like CD8^+^ T Lymphocytes and CD8^+^ T Lymphocytes Responses

**DOI:** 10.3389/fimmu.2016.00411

**Published:** 2016-10-06

**Authors:** Emilia Pachulec, Vanessa Neitzke-Montinelli, João P. B. Viola

**Affiliations:** ^1^Program of Cellular Biology, Brazilian National Cancer Institute (INCA), Rio de Janeiro, Brazil

**Keywords:** NFAT2, CD8^+^ T lymphocytes, innate-like CD8^+^ T cells, PLZF, IFN-γ

## Abstract

Nuclear factor of activated T cells (NFAT) 2 null mutant mice die *in utero* of cardiac failure, precluding analysis of the role of NFAT2 in lymphocyte responses. Only the NFAT2^−/−^/Rag-1^−/−^ chimeric mice model gave insight into the role of NFAT2 transcription factor in T lymphocyte development, activation, and differentiation. As reports are mainly focused on the role of NFAT2 in CD4^+^ T lymphocytes activation and differentiation, we decided to investigate NFAT2’s impact on CD8^+^ T lymphocyte responses. We report that NFAT2 is phosphorylated and inactive in the cytoplasm of naive CD8^+^ T cells, and upon TCR stimulation, it is dephosphorylated and translocated into the nucleus. To study the role of NFAT2 in CD8^+^ T responses, we employed NFAT2^fl/fl^CD4-Cre mice with NFAT2 deletion specifically in T cells. Interestingly, the absence of NFAT2 in T cells resulted in increased percentage of non-conventional innate-like CD8^+^ T cells. These cells were CD122^+^, rapid producer of interferon gamma (IFN-γ) and had characteristics of conventional memory CD8^+^ T cells. We also observed an expansion of PLZF^+^ expressing CD3^+^ thymocyte population in the absence of NFAT2 and increased IL-4 production. Furthermore, we found that CD8^+^ T lymphocytes deficient in NFAT2 had reduced activation, proliferation, and IFN-γ and IL-2 production at suboptimal TCR strength. NFAT2 absence did not significantly influence differentiation of CD8^+^ T cells into cytotoxic effector cells but reduced their IFN-γ production. This work documents NFAT2 as a negative regulator of innate-like CD8^+^ T cells development. NFAT2 is required for complete CD8^+^ T cell responses at suboptimal TCR stimulation and regulates IFN-γ production by cytotoxic CD8^+^ T cells *in vitro*.

## Introduction

CD8^+^ T cells are the key mediators in cell-mediated immunity against intracellular bacteria, viral infections, and tumor cells. Conventional CD8^+^ T cells develop in the thymus from a bone marrow-derived multipotent T cells progenitor through a series of defined and coordinated developmental stages of differentiation, positive and negative selection, and maturation. Mature single positive (SP) CD8^+^ T cells leave the thymus and migrate to peripheral lymphoid organs. In the peripheral lymphoid organs, naive CD8^+^ T cells, which are activated by specific antigens, differentiate into effector T cells that are able to kill target cells through the release of cytotoxic granules, secretion of cytokines, such as interferon gamma (IFN-γ) and Fas/FasL-dependent pathway. A small number of activated CD8^+^ T cells develop into memory T cells that provide long-term protection against reinfection (1). There are several subsets of memory CD8^+^ T cells, and among them, central and effector memory T cells (2). Central memory T cells express high level of CD62L and reside mainly in peripheral lymphoid organs, whereas effector memory T cells express low level of CD62L and are present mainly in non-lymphoid tissues. The development of effector or memory CD8^+^ T cells is dependent on the environment created by the innate immune system, for example, the presence of cytokines such as IL-12 promoting effector CD8^+^ T cells differentiation (3) or IL-7 and IL-15 required for the maintenance and survival of memory CD8^+^ T cells (4).

In addition to conventional CD8^+^ T cells, there are also several subsets of CD8^+^ T cells that acquire functions as a result of their maturation process rather than through antigen activation. These cells are believed to be selected by hematopoietic cells and are dependent on signals transduced through signaling lymphocyte activation molecule (SLAM) family members (5, 6). A subset of these cells, called memory-like or innate-like T cells, has been described in a few gene knockout animal models (5–14) and humans (15). These cells share characteristics with conventional memory CD8^+^ T cells, such as high expression of CD44 and CD122 (5, 6), and with innate cells as they can rapidly secrete IFN-γ upon stimulation (16). It has been reported that memory-like CD8^+^ T cells play important roles in the innate response against infections such as *Listeria monocytogenes*, chronic infections with viruses such as Herpes virus (17–19), and tumor cell lines *in vitro* (20).

Nuclear factor of activated T cells (NFAT) was originally described as a transcription factor inducing the expression of interleukin 2 (IL-2) (21). The NFAT family of transcription factors consists of five members, named NFAT1–5, and the main forms expressed in T cells are NFAT1 and NFAT2 (22). NFAT1 is constitutively expressed in T cells (23), whereas NFAT2 is induced upon T-cell receptor stimulation (24). NFAT proteins reside phosphorylated in the cytoplasm. In activated lymphocytes, NFAT is dephosphorylated by calcineurin (25–28), translocates from the cytoplasm into the nucleus (29–31), where coupled with other transcription factors (26, 32) binds to the promotor regions of multiple genes to induce their transcription. Previous studies showed that NFAT proteins play regulatory roles during T-cell differentiation and effector functions. NFAT1 deficiency in T cells diminished Th1 differentiation and induced IL-4 production (33). NFAT1 was also reported to contribute to IL-21 expression and to limit the immunosuppressive function of CD4^+^CD25^+^Foxp3^+^GITR^+^ T regulatory (Treg) cells (34).

The role of NFAT2 in T-cell differentiation is not fully understood, as the total inactivation of NFAT2 gene in mice led to an early death of mice embryos (35). Previous analysis on Th1- and Th2-skewed T cells isolated from NFAT2^−/−^/Rag-1^−/−^ chimeric mice revealed an involvement of NFAT2 in the induction of the Th2-cytokines IL-4 and IL-6, whereas it had no effect on IFN-γ and IL-2 expression in Th1 cells (36–38). NFAT2 binding sites were found within the *Il17a* promoter (39) and the *Il10* promoter (40). Recently, NFAT2 has been shown as a positive regulator of RORγT and Th17 cytokines during TGF-β-mediated Th17-cell differentiation (41). NFAT2-deficient TGF-β-induced iTreg cells showed a slight reduction of CD25 and Foxp3 expression as compared to WT cells (42), indicating no essential role for NFAT2 in iTreg cell development. Up to now, most of the available studies are focused on the role of NFAT2 in CD4^+^ T lymphocytes differentiation and little is known about its function in CD8^+^ T lymphocytes responses.

In this study, we analyzed the role of NFAT2 in CD8^+^ T cell development and differentiation with the help of conditional NFAT2-deficient mice that were generated by crossing NFAT2^fl/fl^ mice to CD4-Cre mice. These mice show a functional NFAT2 deficiency beyond double positive (DP) thymocytes, consequently CD8^+^ mature T cells. Our results indicate that NFAT2 plays an important role in the development of innate-like CD8^+^ T cells in the thymus. We further demonstrate that conditional inactivation of NFAT2 in T cells alter the threshold of CD8^+^ T cell activation, proliferation, and cytokines production but not differentiation. NFAT2 is not essential for differentiation into effector CD8^+^ T lymphocytes *in vitro*; however, it is important for IFN-γ production by cytotoxic CD8^+^ T cells.

## Materials and Methods

### Animals

C57BL/6 and NFAT2^fl/fl^CD4-Cre were bred and maintained at the Brazilian National Cancer Institute (INCA) animal facility (Rio de Janeiro, RJ, Brazil). Also, 6- to 12-week-old mice were used in all experiments. NFAT2^fl/fl^CD4-Cre mice were generated in Dr. Anjana Rao’s laboratory (La Jolla Institute for Allergy and Immunology, San Diego, CA, USA). All animal experiments were performed in accordance with the Brazilian Government’s ethical and animal experimental regulations. The experiments were approved and conducted according to the animal welfare guidelines of the Ethics Committee of Animal Experimentation from INCA (CEUA process no. 004/13).

### Cell Culture

Primary cells were cultured in DMEM supplemented with 10% FCS, l-glutamine, streptomycin–penicillin, essential and non-essential amino acids, vitamins, HEPES, and 2-ME (all from Gibco) in a humidified environment containing 5% CO_2_ at 37°C. P815 cell line was cultured in RPMI supplemented with 10% FCS, l-glutamine, streptomycin–penicillin, sodium pyruvate, and 2-ME (all from Gibco). CD8^+^ T cells were purified from total lymph nodes (inguinal, brachial, and axillary). Cells were isolated with Dynal Mouse CD8 Negative Isolation Kit (Invitrogen). For cytometric purity analysis, cells were stained with anti-CD4-PE and anti-CD8-FITC Abs (both from BD Pharmingen) and analyzed by flow cytometry on a FACScan (BD Biosciences). Cell populations were isolated to >95% purity. Total lymph nodes or purified CD8^+^ T lymphocytes were activated *in vitro* for indicated times with plate-bound anti-CD3 (1 μg/ml; BD Pharmingen; otherwise indicated) plus soluble anti-CD28 (1 μg/ml; BD Pharmingen). To differentiate CD8^+^ T lymphocytes into cytotoxic CD8^+^T lymphocytes *in vitro*, 1 × 10^6^ cells were activated for 48 h with plate-bound anti-CD3 (1 μg/ml) plus soluble anti-CD28 (1 μg/ml). Cells were expanded daily for 4 days with 200 U/ml of murine recombinant IL-2 (43). At day 5, cells were analyzed by flow cytometry and in cytotoxicity assay *in vitro*.

### Cytoplasmic and Nuclear Protein Extraction

Purified CD8^+^ T lymphocytes (3 × 10^6^) were stimulated or left untreated for indicated times with plate-bound anti-CD3 plus soluble anti-CD28 (both at 1 μg/ml). To extract cytoplasmic fraction, cells were lysed in buffer containing 10 mM Tris-Cl pH 7.5, 10 mM NaCl, 3 mM MgCl_2_, 0.5 mM DTT, 0.1 mM EDTA, 0.5% NP-40, 1 mM PMSF, and protease inhibitor. Nuclear extracts were obtained by lysis of the nuclei with buffer containing 40 mM Tris-Cl pH 7.5, 10 mM EDTA, 60 mM sodium pyrophosphate, and 5% SDS followed by incubation at 100°C for 15 min.

### Western Blot

Total protein extract from 3 × 10^6^ T lymphocytes was obtained from cells lysis in buffer containing 40 mM Tris pH 7.5, 60 mM sodium pyrophosphate, 10 mM EDTA, and 5% SDS, followed by incubation at 100°C for 15 min. Total cell lysates were resolved by SDS-PAGE, and the separated proteins were transferred onto a nitrocellulose membrane. The antibodies used were GAPDH monoclonal antibody 6C5 (Santa Cruz Biotechnology, Santa Cruz, CA, USA) and NFAT2 monoclonal antibody 7A6 (Santa Cruz Biotechnology, Santa Cruz, CA, USA). The immunodetection was performed with the ECL Western Blotting Detection Kit (GE Healthcare).

### Immunofluorescence

Intracellular localization of NFAT2 protein was analyzed in purified CD8^+^ T cells (5 × 10^5^ cells) from C57BL/6 mice by immunofluorescence staining. Briefly, cells were stimulated or left unstimulated for indicated time with plate-bound anti-CD3 plus soluble anti-CD28 (both at 1 μg/ml). Then, cells were fixed in 4% paraformaldehyde, permeabilized with 0.5% Non-idet P-40, and stained with anti-NFAT2 Ab. The cells were photographed with Confocal Laser Scanning Microscope FV10i-O Olympus.

### Flow Cytometry

To analyze cell surface expression levels of the various markers, the cell suspensions (1 × 10^6^) were incubated with following mAbs: anti-CD3-APC, anti-B220-FITC, anti-CD4-PE, anti-CD4-FITC, anti-CD8-FITC, anti-NK1.1-PerCP.Cy5.5, anti-PLZF-PE, anti-CD44-FITC, anti-CD69-APC, anti-CD62L-PE, anti-CD62L-PerCP, anti-CD25-APC, anti-CD122-PE, and anti-CD127-PE (all BD Pharmingen) acquired in a FACScan (Becton Dickinson, Mountain View, CA, USA) and analyzed using FlowJo software.

### Intracellular Staining

For intracellular cytokine staining, 1 × 10^6^ cells were stimulated *in vitro* for 6 h with PMA (10 nM) plus ionomycine (1 μM, both from Calbiochem). Brefeldin A (1:1000; BD Pharmingen) was added to the culture for last 2 h. Cells were harvested and stained with anti-CD8-FITC Abs. Then, cells were fixed, permeabilized, and stained with anti-IFN-γ-FITC, anti-IL-2-PE, anti-IL-4-APC, and anti-Granzime B-FITC Abs. For PLZF intracellular staining, cells were harvested and stained with anti-CD3-APC, fixed, permeablized, and stained with anti-PLZF-PE. Samples were analyzed by flow cytometry on a FACScan (BD Biosciences) and FlowJo software.

### Proliferation Assay

Purified CD8^+^ T lymphocytes (5 × 10^6^) were stained with CFSE Cell Proliferation Assay (Invitrogen) according to manufacturer’s instructions, then stimulated or not with plate-bound anti-CD3 (0.25 μg/ml) plus soluble anti-CD28 (1 μg/ml) for indicated times in the absence or presence of 200 U/ml of IL-2. Carboxyfluorescein diacetate succinimidyl ester (CFSE) dilution was analyzed by flow cytometry on FACScan (BD Biosciences) and FlowJo Software.

### RNA Extractions and Real-time RT-PCR

Total RNA from total or sorted into CD44^low^ and CD44^high^ naive CD8^+^ T lymphocytes, total thymocytes, CD4^+^ thymocytes, and total splenocytes was extracted using Trizol LS Reagent (Invitrogen), and first-strand cDNA was synthesized using ImProm-II Reverse Transcription System (Promega) or Superscript II kit (Life Technology) for sorted CD44^low^ and CD44^high^ CD8^+^ T lymphocytes and CD4^+^ thymocytes. Real-time polymerase chain reactions for NFAT2 and PLZF were performed using SYBR Green master mix (Applied Biosystems) and for Blimp-1, Tbx21, IL-4, Eomes using Taqman probes. Actin or HPRT were used as endogenous control. Sequences of primers used for SyberGreen real-time PCR are NFAT2-F 5′-CCAGAAAATAACATGCGAGCC-3′, NFAT2-R 5′-GTGGGATGTGAACTCGGAAG-3′, PLZF-F 5′-GAGCAGTGCAGCGTGTGT-3′, and PLZF-R 5′-AACCGTTTTCCGCAGAGTT-3′ (44), Actin-F 5′-ATGGTGGGAATGGGTCAGAAG-3′, Actin-R 5′-TTCTCCATGTCGTCCCAGTTG-3′. All procedures were performed according to the manufacturers’ instructions.

### *In Vitro* Cytotoxicity Assay

Target cells (P815) at 10^6^/ml were labeled with 10-μM Calcein-AM (Molecular Probes) in complete medium for 30 min at 37°C. The assay was performed in V bottom 96-well microtiter plates with E:T ratios ranging from 2:1 to 0.5:1 with wells for spontaneous (only target cells in complete medium) and maximum release (only target cells in medium plus 2% Triton X-100) in the presence of 1 μg/ml of soluble anti-CD3, in triplicate. Each well contained from 1 × 10^5^ to 2.5 × 10^4^ lymphocytes in 100 μl of complete medium and 5 × 10^4^ target cells/50 μl of complete medium. After incubation at 37°C in 5% CO_2_ for 4 h, 75 μl of each supernatant was harvested and transferred into new plates. Samples were measured using a Spectramax Gemini dual-scanning microplate spectrofluorimeter (Molecular Devices) (excitation: 485 nm; emission: 530). Data were expressed as arbitrary fluorescent units (AFU). Percent lysis was calculated with the following formula: [(A sample − A spontaneous)/(A max − A spontaneous)] × 100.

### Statistical Analysis

For single comparisons, unpaired Student’s *t*-test was used. Flow cytometry and mean fluorescence intensity analysis was performed using FlowJo 10.1r5 software and analyzed for statistical significance by unpaired two-tailed Student’s *t-*test using Prism (GraphPad Software). Differences with *p* < 0.05 were considered statistically significant.

## Results

### NFAT2 Is Present and Functional in CD8^+^ T Lymphocytes

TCR stimulation results in calcium mobilization and consequent activation of NFAT transcription factors. NFAT is activated by the phosphatase calcineurin, which dephosphorylates multiple phosphoserines in the regulatory domain of NFAT, leading to NFAT nuclear translocation (45).

**Figure 1:**
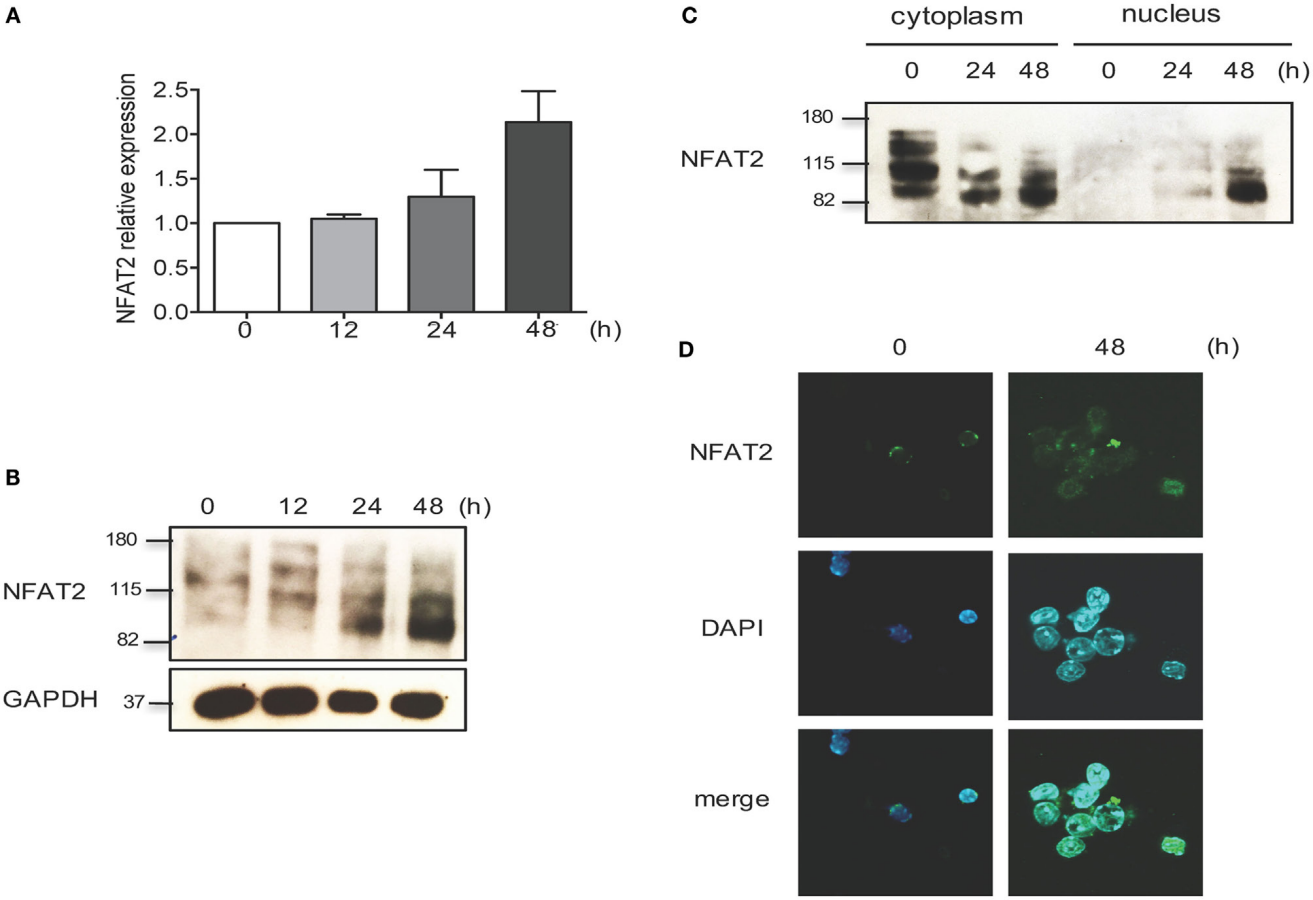
**NFAT2 is present and functional in CD8^+^ T lymphocytes**. CD8^+^ T lymphocytes were purified from naive C57BL/6 mice, as described, and then left unstimulated (0) or stimulated *in vitro* with anti-CD3 plus anti-CD28 (both at 1 μg/ml) for indicated times. **(A)** Total RNA was isolated, and NFAT2 mRNA levels were analyzed by real-time RT-PCR assay using SYBR green master mix. The data are normalized to the β-actin levels. Data are shown as mean ± SD of three independent experiments. Detection of NFAT2 transcription factor in CD8^+^ T cells in total lysates **(B)** and in cytoplasmic and nuclear fractions **(C)** by Western blot. **(D)** Cellular localization of NFAT2 protein in CD8^+^ T cells by immunofluorescence staining. All data are representative of at least two independent experiments.

To evaluate the NFAT2 expression and activation, CD8^+^ T lymphocytes from WT mice were purified, left unstimulated, or stimulated *in vitro* with anti-CD3 plus anti-CD28 for different times. As expected, TCR stimulation increased both NFAT2 mRNA and protein level (Figures 1A,B). In unstimulated cells, NFAT2 was present in the cytoplasm in an inactive phosphorylated form (Figures 1C,D). At 24 h post-activation, we observed NFAT2 dephosphorylation (Figures 1B,C), and at 48 h post-activation, we detected NFAT2 in the nucleus (Figures 1C,D). These data show that NFAT2 protein is present and functional in CD8^+^ T lymphocytes.

### Development of Innate-Like CD8^+^ T Cells in the Absence of NFAT2

**Figure 2:**
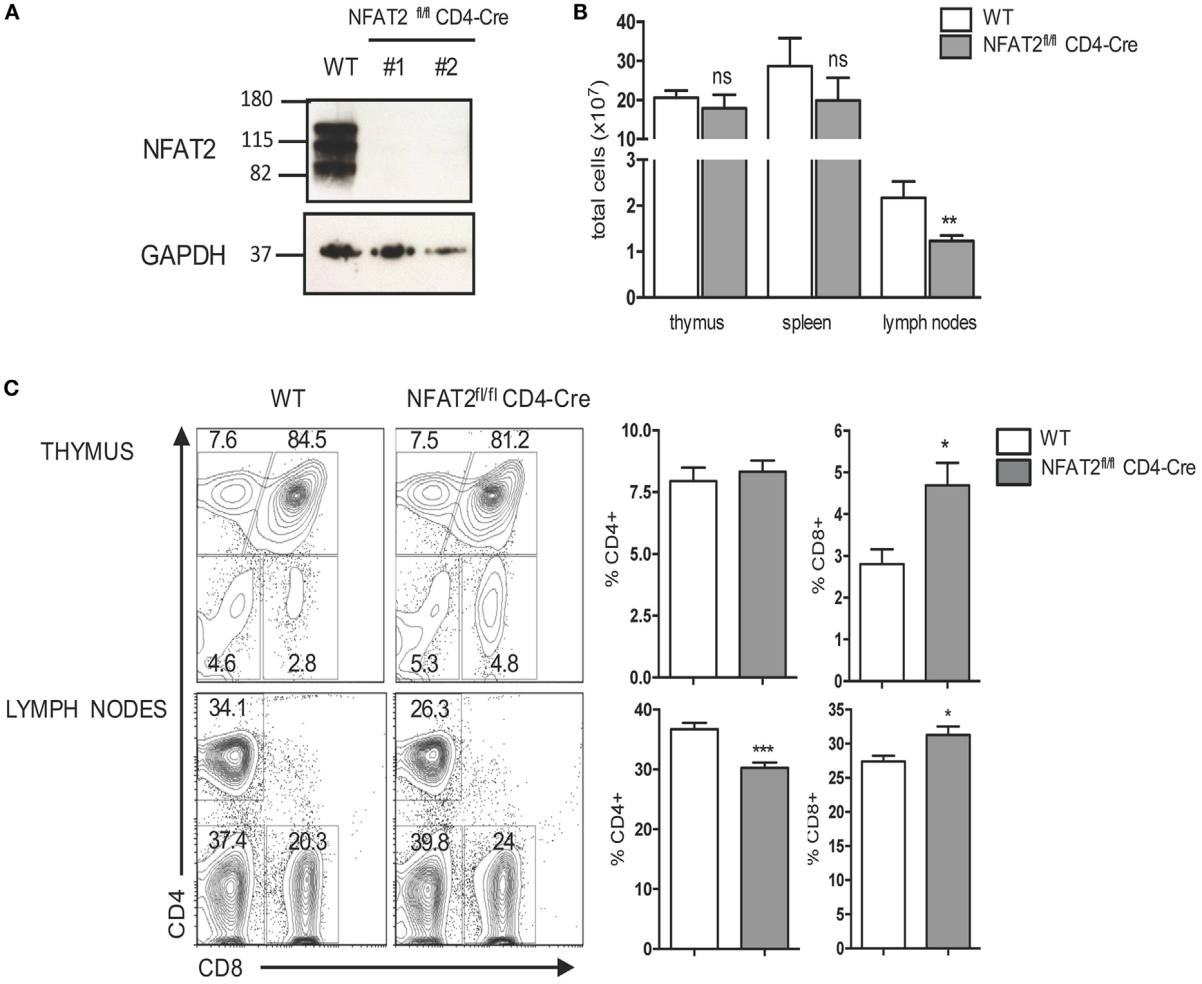
**NFAT2 deficiency increases the frequency of CD8^+^ T cells**. **(A)** Detection of NFAT2 transcription factor in CD8^+^ T cells’ total protein lysates from WT and NFAT2^fl/fl^CD4-Cre mice by Western blot. **(B)** Thymus, spleens, and lymph nodes were harvested from WT or NFAT2^fl/fl^CD4-Cre mice, and the total number of cells were counted using Trypan blue exclusion. **(C)** Flow cytometric analysis of thymocytes and total lymph nodes cells from WT or NFAT2^fl/fl^ CD4-Cre mice. Shown is the frequency of CD4- and CD8-expressing cells. Right, the percentage of CD4^+^ and CD8^+^ cells for all the mice. All results are representative of at least two independent experiments. All data are shown as mean ± SD. The ns indicates not significant, *indicates *p* < 0.05, and **indicates *p* < 0.01 compared to WT mice.

To study how NFAT2 regulates CD8^+^ T cell development and responses, we used mice with T cell-specific deficiency in NFAT2. We used CD4-*cre* mice crossed to mice with NFAT2 flanked by *loxP* sites (NFAT2^fl/fl^). In this model, the NFAT2 gene is excised at the DP stage of thymocyte development, affecting both SP CD4 and CD8 mature T cells. The absence of NFAT2 protein expression in CD8^+^ T cells from NFAT2^fl/fl^CD4-Cre mice was confirmed by Western blot (Figure 2A).

NFAT2^fl/fl^CD4-Cre mice showed only little changes in total cell numbers in the thymus and spleen, but a significant decrease of total cell number in lymph nodes (Figure 2B), and consistently NFAT2^fl/fl^CD4-Cre mice had reduced size of lymph nodes (data not shown). Thymus of NFAT2^fl/fl^CD4-Cre mice had similar frequency of DP as well as CD4^+^ single positive (SP) thymocytes as WT (Figure 2C). Interestingly, there was an increase in the frequency of CD8^+^ SP thymocytes (Figure 2C). To examine whether observed difference was thymus-specific or a general characteristic, we then analyzed spleen and lymph nodes of NFAT2^fl/fl^CD4-Cre mice. The spleen of NFAT2^fl/fl^CD4-Cre mice had significant reduction of the frequency of total T lymphocytes, in particular CD4^+^, and a slight increase of B lymphocytes, while no change was observed for CD8^+^ T cells (data not shown). Lymph nodes of NFAT2^fl/fl^CD4-Cre mice showed significantly lower number of CD8^+^ T lymphocytes in NFAT2^fl/fl^CD4-Cre mice compared to WT (data not shown), however, with only little changes in the frequency of total T and B lymphocytes. Similarly, to the spleen, we observed a decrease in the frequency of CD4^+^ T cells (Figure 2C), and as seen in the thymus, an increase in the frequency of CD8^+^ T lymphocytes (Figure 2C).

**Figure 3:**
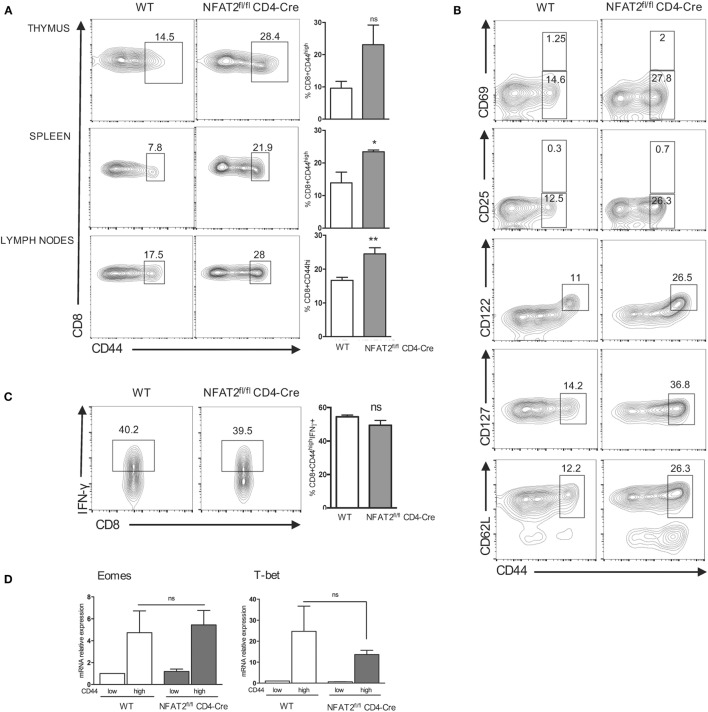
**NFAT2 deficiency leads to the development of innate-like CD8^+^ T cells**. **(A)** Flow cytometric analysis CD8^+^ T cells for CD44 expression. Right, the percentage of CD8^+^CD44^high^ cells for all the mice. **(B)** Total CD8^+^ T cells were analyzed for the expression of CD69, CD25, CD122, CD127, and CD62L. Shown are the percentages of T CD8^+^CD44^high^ populations expressing or not specific surface markers. **(C)** Total CD8^+^ T cells were stimulated with PMA plus ionomycin for 6 h, and cells in CD8^+^CD44^high^ gate were analyzed for IFN-γ production. Right, the percentage of CD8^+^CD44^high^IFN-γ^+^ cells for all the mice. **(D)** Total RNA was isolated from CD8^+^CD44^high^ and CD8^+^CD44^low^ populations from lymph nodes of WT and NFAT2^fl/fl^CD4-Cre mice, and Tbx21 and Eomes mRNA levels were analyzed by real-time RT-PCR assay using Taqman probe. The data are normalized to the HPRT levels. All results are representative of at least two independent experiments. All data are shown as mean ± SD. The ns indicates not significant, *indicates *p* < 0.05, and **indicates *p* < 0.01 compared to WT mice.

A higher proportion of CD8^+^ T cells in the thymus and lymph nodes of NFAT2^fl/fl^CD4-Cre mice compared to WT was expressing CD44, an activated T cell marker. An increase in the frequency of CD8^+^CD44^high^ cells was also observed in the spleen of NFAT2^fl/fl^CD4-Cre mice even without a significant increase in the frequency of CD8^+^ T cells (Figure 3A). As the percentage of CD8^+^CD44^high^ cells was increased in the thymus, lymph nodes, and spleen, it suggested that this is not dependent on preferential enrichment of these cells in specific tissues.

To exclude the fact that CD8^+^CD44^high^ T cells are present due to an activation, we examined the expression of two activation markers: CD69 (early activation marker) and CD25 (IL-2 receptor α-chain). CD8^+^CD44^high^ population from both WT and NFAT2^fl/fl^CD4-Cre mice expressed low and similar percentage of CD69^+^ and CD25^+^ cells showing that CD8^+^CD44^high^ population is not pre-activated *in vivo* (Figure 3B).

Expression of CD122, the IL-2Rβ chain, has been reported to define the population of innate-like CD8^+^ T cells (46), and we found that the CD8^+^CD44^high^ population in both WT and NFAT2^fl/fl^ CD4-Cre mice expressed CD122, although the NFAT2^fl/fl^CD4-Cre mice carried larger percentage of these cells (Figure 3B).

Moreover, NFAT2^fl/fl^CD4-Cre mice had larger percentage of CD8^+^CD44^high^ CD127^+^ and CD8^+^CD44^high^ CD62L^+^ compared to WT (Figure 3B), further showing that CD8^+^CD44^high^ population display conventional memory T cell characteristics without previous antigen exposure.

Another characteristic of innate-like CD8^+^ T cells is the rapid production of large amount of IFN-γ upon stimulation (16). To determine if these innate-like CD8^+^ cells exhibit similar functional behavior, we analyzed their ability to rapidly secrete IFN-γ upon stimulation. Comparison of IFN-γ production by PMA plus ionomycin stimulated CD8^+^CD44^high^ T cells *in vitro* indicated that NFAT2-deficient cells behave similarly to those from WT mice in rapid (within 6 h) secretion of this cytokine. A similar percentage of the CD8^+^CD44^high^ T cells responded from both WT and NFAT2^fl/fl^CD4-Cre mice (Figure 3C). By contrast, CD8^+^CD44^low^ T cells did not secrete any appreciable IFN-γ in this time period (data not shown).

Further analysis of CD8^+^CD44^high^ population revealed that cells from both NFAT2^fl/fl^CD4-Cre and WT mice expressed elevated *Tbx21* and *Eomes* mRNA level compared to respective CD8^+^CD44^low^ counterparts. *Eomes* expression remained similar (Figure 3D), while *Tbx21* mRNA was reduced without a significant difference in CD8^+^CD44^high^ cells from NFAT2^fl/fl^CD4-Cre animals compared to WT.

Taken together, these results demonstrate that NFAT2 deficiency leads to the development of innate-like CD8^+^ T cells without affecting theirs characteristics, such as surface markers and transcription factors expression and IFN-γ production.

### NFAT2 Deficiency in T Cells Results in the Expansion of PLZF^+^ T Cells

**Figure 4:**
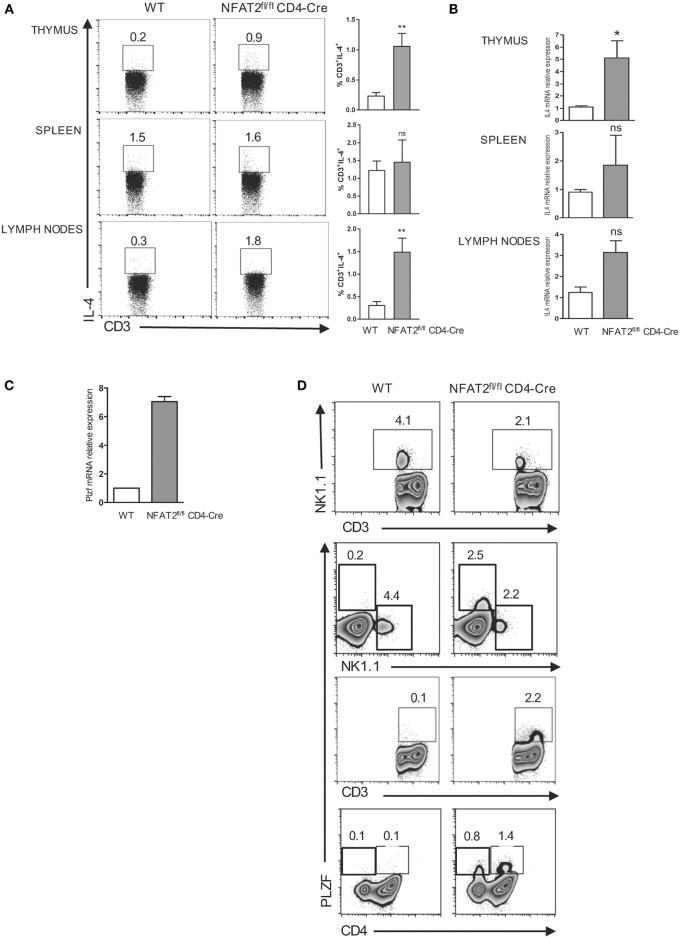
**Expansion of PLZF expressing cells in the absence of NFAT2**. Total thymocytes, splenocytes, and lymph nodes cells were stimulated with PMA plus ionomycin for 6 h. IL-4-producing cells were analyzed in CD3^+^ gate. Right, the percentage of CD3^+^IL-4^+^ cells for all the mice **(A)**. Total RNA was isolated from thymus, spleen, and lymph nodes from WT and NFAT2^fl/fl^CD4-Cre mice, and IL-4 mRNA levels were analyzed by real-time RT-PCR assay using Taqman probe. The data are normalized to the HPRT levels **(B)**. Total RNA was isolated from CD4^+^ thymocytes and analyzed for PLZF mRNA level by real-time RT-PCR using SyberGreen. The data are normalized to the actin levels **(C)**. Flow cytometric analysis of CD3^+^, CD4^+^, and NK1.1^+^ thymocytes for PLZF expression **(D)**. All results are representative of at least two independent experiments. All data are shown as mean ± SD. The ns indicates not significant, *indicates *p* < 0.05, and **indicates *p* < 0.01 compared to WT mice.

Interleukin-4 is a cytokine classically associated with CD4^+^ T helper type 2 differentiation (47) but has been recently shown in different mice models to be also required for the development of innate-like CD8^+^ T lymphocytes through Eomes upregulation (9–11, 44, 48–50). To compare the IL-4 production between NFAT2^fl/fl^CD4-Cre and WT mice, we stimulated WT and NFAT2-deficient thymocytes, splenocytes, and total lymph nodes cells *ex vivo* with PMA plus ionomycine for 6 h. We analyzed the production of IL-4 by CD3^+^ cells. We observed that NFAT2^fl/fl^CD4-Cre mice had higher percentage of CD3^+^IL-4^+^ cells in the thymus and lymph nodes and only small increase in the spleen (Figure 4A). Consistently, IL-4 mRNA was clearly more abundant in the thymus, lymph nodes, and even the spleen of NFAT2^fl/fl^CD4-Cre mice (Figure 4B).

In several gene-deficiency models, IL-4 is produced by an expanded population of T cells expressing the transcription factor PLZF. First, we tested whether NFAT2 absence affects the PLZF expression, by measuring the PLZF mRNA expression in the thymus by real-time PCR. Indeed, we found much higher PLZF mRNA level in NFAT2-deficient CD4^+^ thymocytes compared to WT (Figure 4C). Interestingly, in NFAT2^fl/fl^CD4-Cre mice, we observed the reduction of CD3^+^ NK1.1^+^ as compared to WT. Further analysis showed that PLZF^+^ T cell population in NFAT2^fl/fl^CD4-Cre mice was NK1.1^−^, CD4^+^, or CD4^−^ (Figure 4D). Thus, the absence of NFAT2 results in the expansion of PLZF^+^, NK1.1^−^, CD4^+^, or CD4^−^ expressing T cells and increases the IL-4 production in the thymus and the periphery, which, in consequence, may lead to the higher frequency of innate-like CD8^+^ T cells.

### The Absence of NFAT2 Increases the Threshold of T Cell Responses

**Figure 5:**
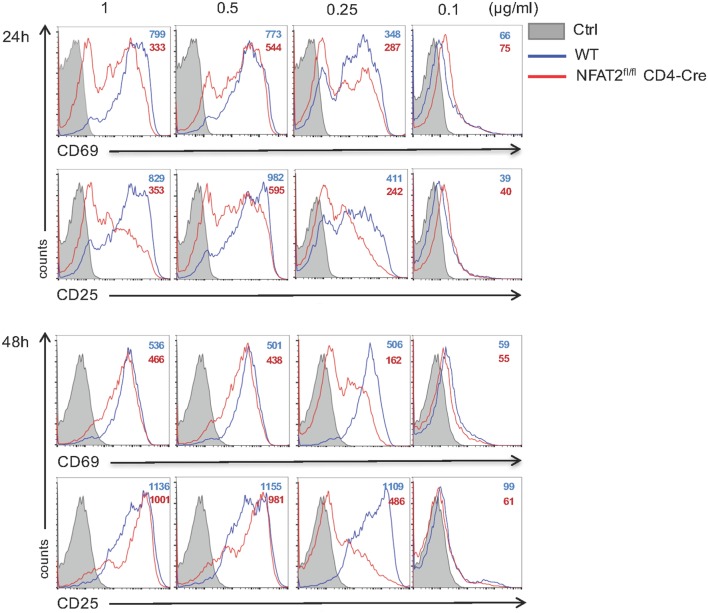
**NFAT2 absence increases the threshold of CD8^+^ T cells activation**. Total lymph nodes cells from WT and NFAT2^fl/fl^CD4-Cre mice were activated for 24 and 48 h with different doses of anti-CD3 and 1 μg/ml of anti-CD28. Cells in CD8^+^ gate were analyzed for the expression of CD69 and CD25. All data are representative of at least three independent experiments.

Nuclear factor of activated T cell transcription factors are ubiquitous regulators of gene expression during cellular activation; however, most reports are focused mainly on CD4^+^ T lymphocytes [for review, see Ref. (23)]. To evaluate the specific role of NFAT2 in CD8^+^ T cell activation, we purified CD8^+^ T lymphocytes from lymph nodes of WT and NFAT2^fl/fl^CD4-Cre mice, and we left them either unstimulated or stimulated for 24 and 48 h with different doses of anti-CD3 plus 1 μg/ml of anti-CD28. As expected, more than 85% of unstimulated cells were positive for CD62L, a protein highly expressed on naive cells, and only few expressed CD69 and CD25 activation markers (data not shown). TCR stimulation with 1, 0.5, and 0.25 μg/ml of anti-CD3 for 24 h led to a reduction of both CD69 and CD25 expression in NFAT2-deficient cells compared to WT (Figure 5, upper panel). The dose of 0.1 μg/ml of anti-CD3 was not sufficient to activate CD8^+^ T cells (Figure 5). Interestingly, TCR stimulation with 1 and 0.5 μg/ml of anti-CD3 for 48 h resulted in a similar cell activation as shown by CD69 and CD25 expression between WT and NFAT2-deficient cells (Figure 5, lower panel), indicating that NFAT2 absence was compensated by another transcription factor, likely NFAT1. Only at lower anti-CD3 concentration (0.25 μg/ml), the cell activation of NFAT2-deficient cells remained significantly reduced in comparison to WT (Figure 5, lower panel), indicating that the absence of NFAT2 increases the threshold of CD8^+^ T cell activation at suboptimal TCR stimulation.

**Figure 6:**
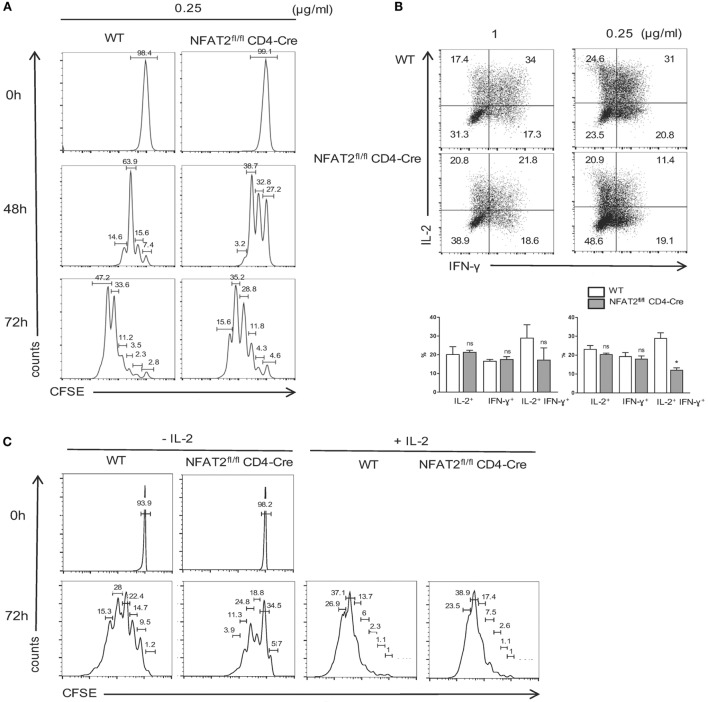
**NFAT2 deficiency increases the threshold of CD8^+^ T cells proliferation and cytokines production**. **(A)** CD8^+^ T cells from lymph nodes of WT and NFAT2^fl/fl^CD4-Cre mice were purified, stained with CFSE as described, and activated with 0.25 μg/ml of anti-CD3 plus 1 μg/ml of anti-CD28 for indicated times. At each time point, CFSE dye dilution was analyzed by flow cytometry. **(B)** CD8^+^ T cells from lymph nodes of WT and NFAT2^fl/fl^CD4-Cre mice were purified and activated for 48 h with either 1 or 0.25 μg/ml of anti-CD3 plus 1 μg/ml of anti-CD28. Then, cells were restimulated with PMA plus ionomycin for 6 h, and intracellular IL-2 and IFN-γ production was analyzed by flow cytometry. Shown is the percentage of IL-2- and IFN-γ-expressing cells. **(C)** CD8^+^ T cells from lymph nodes of WT and NFAT2^fl/fl^CD4-Cre mice were purified, stained with CFSE as described, and activated with 0.25 μg/ml of anti-CD3 plus 1 μg/ml of anti-CD28 in the absence or presence of 200 U/ml IL-2 for indicated times. At each time point, CFSE dye dilution was analyzed by flow cytometry. All data are representative of at least two independent experiments.

As NFAT2 deficiency in CD8^+^ T cells increased the threshold of T cell activation, we next tested the effect of NFAT2 absence on CD8^+^ T cell proliferation. Purified CD8^+^ T cells were stained with CFSE and then activated for different times with 0.25 μg/ml anti-CD3 plus 1 μg/ml of anti-CD28. At indicated times, cell proliferation was monitored by flow cytometric measurement of CFSE dye dilution. Low concentration of anti-CD3 led to a reduced proliferation of NFAT2-deficient CD8^+^ T cells with division indexes of 3.19 for WT and 2.64 for NFAT2-deficient cells after 72 h, indicating that NFAT2 absence increases the threshold of triggering CD8^+^ T cell into division (Figure 6A).

Consistent with defects in cell activation and proliferation, NFAT2-deficient CD8^+^ T cells were also weak producers of both IFN-γ and IL-2. The slight difference was observed even at strong TCR stimulation, but clear decrease of the percentage of IFN-γ- and IL-2-producing cells was observed at suboptimal dose of anti-CD3 (Figure 6B). Next, we decided to determine whether defect of IL-2 production in NFAT2-deficient CD8^+^ T cells results in delayed cell proliferation. Purified CD8^+^ T cells were stained with CFSE and then activated for different times with 0.25 μg/ml anti-CD3 plus 1 μg/ml of anti-CD28 in the absence or presence of 200 U/ml of IL-2. At indicated times, cell proliferation was monitored by flow cytometric measurement of CFSE dye dilution. Addition of IL-2 restored the proliferation of NFAT2-deficient CD8^+^ T cells at 48 h (data not shown) and 72 h post-activation (Figure 6C) demonstrating that reduced proliferation in the absence of NFAT2 is due to decreased IL-2 secretion.

### NFAT2 Regulates IFN-γ Production by Cytotoxic CD8^+^ T Cells

**Figure 7:**
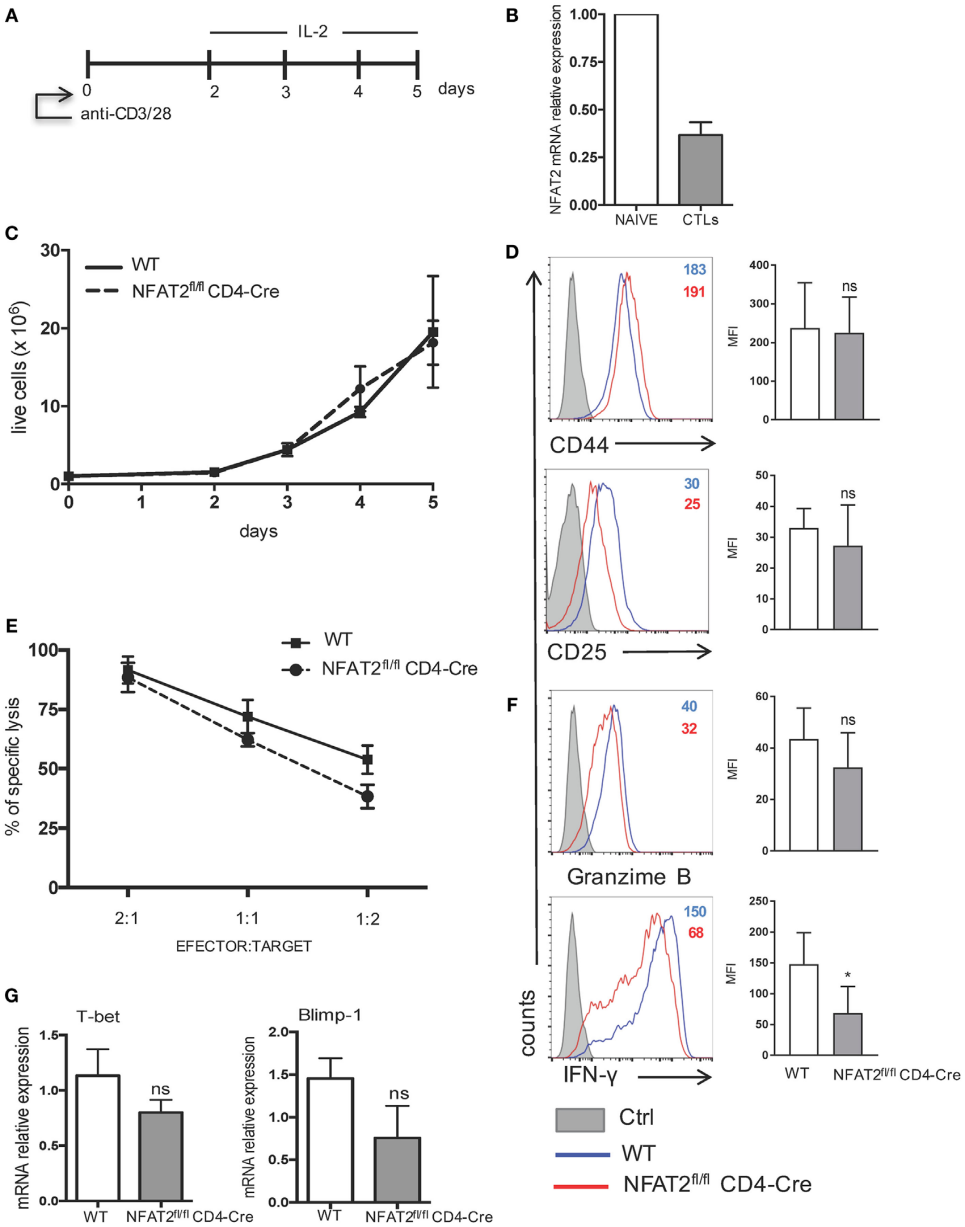
**IFN-γ production by cytotoxic CD8^+^ T cells is regulated by NFAT2**. **(A)** Schematic representation of cytotoxic CD8^+^ T cell differentiation *in vitro*. Purified CD8^+^ T cells from WT and NFAT2^fl/fl^CD4-Cre mice were differentiated *in vitro* into cytotoxic CD8^+^ T lymphocytes as described. **(B)** Total RNA isolated from naive CD8^+^ T cells (NAIVE) or from *in vitro* differentiated cytotoxic CD8+ T lymphocytes (CTLs) were analyzed for NFAT2 mRNA levels by real-time RT-PCR assay using SYBR green master mix. The data are normalized to the β-actin levels. **(C)** Total number of live WT and NFAT2-deficient CD8^+^ T lymphocytes was recorded daily during *in vitro* differentiation using Trypan blue exclusion method. **(D)** Flow cytometric analysis of CD44 and CD25 expression on differentiated cytotoxic WT and NFAT2-deficient CD8^+^ T cells at day 5. The analysis of MFI from three independent experiments on the right. **(E)** At day 5, differentiated cytotoxic CD8^+^ T cells were tested in cytotoxicity assay against P815 cells as described at indicated efector:target ratios or restimulated with PMA plus ionomycin for 6 h, and intracellular granzime B and IFN-γ production was analyzed by flow cytometry. The analysis of MFI from three independent experiments on the right **(F)**. **(G)** Total RNA was isolated from differentiated *in vitro* WT and NFAT2-deficient cytotoxic CD8^+^ T lymphocytes, and T-bet and Blimp-1 mRNA levels were analyzed by real-time RT-PCR assay using Taqman probes. The data are normalized to the HPRT RNA levels. All data are shown as mean ± SD of three independent experiments. The ns indicates not significant and *indicates *p* < 0.05 compared to WT mice.

Antigen-induced CD8^+^ T cells differentiate into cytotoxic CD8^+^ T cells (CTLs) that kill infected cells and release cytokines, such as IFN-γ (1). To analyze if NFAT2-deficient CD8^+^ T cells have any other intrinsic defects besides increased threshold of activation, proliferation, and cytokine secretion, we determined if differentiation and function of CTLs *in vitro* were intact. To differentiate CTLs *in vitro*, we followed as reported (43). Briefly, purified CD8^+^ T cells from WT and NFAT2^fl/fl^CD4-Cre mice were stimulated for 48 h with 1 μg/ml of anti-CD3 plus anti-CD28 and expanded daily with murine recombinant IL-2 (200 U/ml) (Figure 7A). At day 5, cells were collected and analyzed. The WT cytotoxic CD8^+^ T cells differentiated *in vitro* showed a downregulation of NFAT2 mRNA expression compared to naive CD8^+^ T cells (Figure 7B). No significant difference in cell viability and proliferation between WT and NFAT2-deficient CTLs was observed using the Trypan blue exclusion method (Figure 7C). The expression of activation markers, CD44 and CD25, was also comparable between WT and NFAT2-deficient CTLs (Figure 7D), indicating that NFAT2 does not influence either proliferation or activation of cytotoxic CD8^+^ T cells.

CD8^+^ T cytotoxic activity was measured as the ability to effectively lyse P815 cells. NFAT2-deficient CTLs had slightly reduced potential to lyse P815 cells at 1:1 and 1:2 effector:target ratios (Figure 7E). Consistently, granzyme B expression was slightly decreased without statistical significance in NFAT2-deficient CTLs (Figure 7F). Remarkably, NFAT2-deficient CTLs produced significantly less IFN-γ compared to their proficient counterparts (Figure 7F).

Transcription factors T-bet and Blimp-1 are reported to play a crucial role in promoting cytotoxic CD8^+^ T cell differentiation (51, 52); therefore, we tested what is the effect of NFAT2 absence in CTLs on the expression of T-bet and Blimp-1. NFAT2-deficient CTLs had lower expression of both T-bet and Blimp-1, although in both cases, the difference remained insignificant (Figure 7G).

Taken together, these data demonstrate that NFAT2 does not significantly affect the differentiation of CD8^+^ T cells into cytotoxic T cells but positively controls the IFN-γ production.

## Discussion

In this study, we characterized the role of NFAT2 in CD8^+^ T cell development and responses. During analysis of mice lacking NFAT2 in T cells, we noted that these mice in comparison to WT mice had an increased percentage of CD8^+^CD44^high^CD122^+^ cells in the thymus, spleen, and lymph nodes. Upon stimulation with PMA plus ionomycine, CD8^+^CD44^high^ cells rapidly produced IFN-γ; however, in the same manner as WT CD8^+^CD44^high^ indicating that NFAT2 is not essential for IFN-γ production. Rapid IFN-γ production by stimulated naive CD8^+^ T cells was reported to be dependent on NFAT1 (53), thus NFAT2 seems only to regulate the development of CD8^+^CD44^high^CD122^+^ cells.

CD8^+^CD44^high^CD122^+^ population is known as innate-like CD8^+^ T cells, has the phenotype of memory CD8^+^ T cells, and function as innate cells. In contrast to the so-called “true” or “conventional” memory cells that are induced by TCR stimulation with foreign antigen, this population is present in a steady state without contact with the foreign antigen. There have been numerous studies that have investigated innate-like cells in different experimental contexts. Mice deficient in or expressing mutant forms of diverse T-cell signaling molecules or transcription factors, including the scaffold protein SLP-76 (54), Itk (7, 55), the transcription factor KLF2 (Kruppel-like factor 2) (13, 44), the histone acetyltransferase CBP (CREB-binding protein), and the transcriptional regulator Id3 (inhibitor of DNA-binding 3) (12), also display an increased frequency and numbers of innate-like CD8^+^ T cells [reviewed in Ref. (56)]. Similar population of CD8^+^CD44^high^ cells have been reported in mice lacking classical class I molecules, and this phenotype appears to be class 1b restricted (7, 57). Together, the similar phenotypes of these different mouse strains define a network of diverse effector proteins involved in a TCR signaling pathway to gene expression, which together specify increased generation of innate-like CD8^+^ T cells.

Based on the origin, CD8^+^CD44^high^ cells are grouped in two populations. The first is called homeostatic memory or virtual memory that arises both in the lymphopenia and physiological environment in the periphery, in an IL-15-dependent manner (58). CD8^+^CD44^high^ cells appear upon the transfer of naive T cells into lymphopenic mice (44). Consistently, patients recovering from bone marrow transplant, older people, and mice also showed elevated percentages of T cells with a similar phenotype (59–61). In addition, viral and other infections can lead to expanded populations of T cells bearing this phenotype (19).

The second group is IL-4-induced memory cells or innate-like CD8^+^ T cells identified in gene-deficient or mutant mice and also in normal mice (62). A common feature of these mice is the expansion of one or more subsets of PLZF^+^ thymocytes secreting IL-4, which in turn drive the expansion of the innate-like CD8^+^ T cells in a cell-extrinsic manner (10, 11, 44, 48). PLZF^+^ cells are rare or not activated to produce IL-4 in WT mice; however, distinct genetic alterations influence their generation and activation, and consequently increase innate-like CD8^+^ T cells presence. We show that NFAT2 deficiency in T cells results in the expansion of PLZF^+^ NK1.1^−^CD4^+^ and CD4^−^ T cells. Consistently, we also observed increased IL-4 mRNA and IL-4 production by CD3^+^ cells in the thymus, spleen, and lymph nodes of NFAT2^fl/fl^CD4-Cre mice. Therefore, innate-like CD8^+^ T cells development in NFAT2^fl/fl^CD4-Cre mice seem to also be IL-4 dependent. To explain how NFAT2 exactly regulates the development of PLZF^+^ thymocytes and IL-4 production by those cells, further investigation is required.

Both virtual memory and innate CD8^+^ T cells are phenotypically and functionally very similar. They differ in cytokine dependence, IL-15 for virtual memory, and IL-4 for innate-like CD8^+^; origin, arising in the periphery vs. thymus, respectively, and differential expression profile of certain genes, such as *Tnfarsf1a, Nfkb2, Eomes*, and *Bcl2l11* (63). Interestingly, virtual memory CD8^+^ T cells were also reported to develop in the presence of IL-4 overproduction in the periphery (64). It is still unclear whether these cell populations represent different subsets of alternative memory CD8^+^ T cells or if they are the same cell population that acquired its phenotype with different pathways. If we claim two distinct populations of alternative memory CD8^+^ T cells, it is possible that CD8^+^CD44^high^ population in NFAT2^fl/fl^CD4-Cre mice is heterogeneous and is comprised of both innate-like CD8^+^ T cells developed in the thymus and virtual memory CD8^+^ T cells developed in the periphery, both in IL-4-dependent manner.

In the absence of NFAT2, CD8^+^ T cells showed increased threshold for activation, proliferation, and cytokines production under suboptimal TCR stimulation. The fact that CD69 and CD25 activation markers were strongly reduced at 24 h post-activation and then at 48 h post-activation were similar to WT level indicates that NFAT2 deficiency has been compensated by other transcription factor, likely NFAT1. Only at suboptimal TCR stimulation, NFAT2 was crucial for complete CD8^+^ T cell responses.

Nuclear factor of activated T cells 2 deficiency resulted as well in the reduced capacity of CD8^+^ T cells to proliferate under suboptimal anti-CD3 concentration. Previous studies using NFAT2^−/−^/Rag-1^−/−^ chimeric mice likewise reported impaired proliferation of NFAT2-deficient T cells (38) but independently of autocrine IL-2 secretion. In the EAE model, NFAT2 deficiency reduced the proliferation capacity of MOG-specific T cells in IL-2-dependent and -independent manners (41). In our model, CD8^+^ T cells produced less IL-2 under suboptimal TCR stimulation, and NFAT2 deficiency reduced proliferation in IL-2-dependent manner.

Nuclear factor of activated T cells 2 is not required for the cytotoxic activity of CD8^+^ T cells *in vitro* but appears to be important for their IFN-γ production. IFN-γ production by cytotoxic CD8^+^ T cells is regulated by T-bet and Blimp-1 transcription factors. There was only a slight decrease in T-bet expression but clear reduction of Blimp-1 expression in the absence of NFAT2. Whether NFAT2 influences the IFN-γ expression by directly binding to the IFN-γ promoter or the reduction of IFN-γ protein expression is secondary to the downregulation of Blimp-1, and/or T-bet transcription factors have to be further investigated. Similar defect in IFN-γ production was also found in CD8^+^ T cytotoxic cells in EAE model *in vivo* (41). NFAT2^−/−^/Rag-1^−/−^ chimeric mice developed comparable cytotoxic T lymphocyte responses to the virus indicating that NFAT2 is not essential for the helper function *in vivo* (38).

In conclusion, we demonstrate in this study that NFAT2 is involved in several aspects of CD8^+^ T cell biology. In regard to T cell development, NFAT2 is a negative regulator of innate-like CD8^+^ T cells and PLZF^+^ expressing T cells development. What concerns CD8^+^ T cell responses upon TCR stimulation, we show that the absence of NFAT2 increases the threshold of CD8^+^ T cell activation and decreases IFN-γ and IL-2 production and thus delays cell proliferation in an IL-2-dependent manner. Furthermore, we show that although NFAT2 does not impact cytotoxic CD8^+^ T cells differentiation *in vitro*, it is clearly required for IFN-γ production. Finally, we show that NFAT2 regulates both innate immune response *via* innate-like CD8^+^ T cells and adaptive immune response by controlling IFN-γ production by effector CD8^+^ T lymphocytes.

## Author Contributions

EP performed most of the experiments, analyzed the data, contributed to experimental design, and wrote the manuscript; VN-M performed some of the experiments, participated in the discussions, and helped with the CD8^+^ T lymphocytes differentiation and *in vitro* cytotoxicity assay; JV designed and was responsible for the study, assisted with data analysis, and wrote the manuscript.

## Conflict of Interest Statement

The authors declare that the research was conducted in the absence of any commercial or financial relationships that could be construed as a potential conflict of interest.
